# Breathing mode influence in craniofacial development

**DOI:** 10.1016/S1808-8694(15)31304-5

**Published:** 2015-10-20

**Authors:** Fernanda Campos Rosetti Lessa, Carla Enoki, Murilo Fernandes Neuppmann Feres, Fabiana Cardoso Pereira Valera, Wilma Terezinha Anselmo Lima, Mirian Aiko Nakane Matsumoto

**Affiliations:** ^1^ Master studies in Pediatric Dental Sciences, School of Dental Sciences, Ribeirão Preto-USP; ^2^ Ph.D. studies in Pathology, Medical School, Ribeirão Preto- USP; ^3^ Dentist; ^4^ Assistant Physician, Medical School, Ribeirão Preto- USP; ^5^ Faculty Member and Professor of Otorhinolaryngology, Medical School, Ribeirão Preto- USP; ^6^ Professor (PhD), Department of Children's Clinic and Preventive and Social Dental Sciences, School of Dental Sciences, Ribeirão Preto- USP

**Keywords:** craniofacial development, mouth breathers, facial height

## Abstract

**Aim:**

the aim of this study was to evaluate the differences in facial proportions of nose and mouth breathing children using cephalometric analysis.

**Study design:**

transversal cohort.

**Material and Method:**

Sixty cephalometric radiographs from pediatric patients aged 6 to 10 years were used. After otorhinolaryngological evaluation, patients were divided into two groups: Group I, with mouth breathing children and group II, with nose breathers. Standard lateral cephalometric radiographs were obtained to evaluate facial proportions using the following measures: SN.GoGn, ArGo.GoMe, N-Me, N-ANS, ANS-Me and S-Go; and the following indexes: PFH-AFH ratio: S-Go/N-Me; LFH-AFH ratio: ANS-Me/N-Me and UFH-LFH ratio: N-ANS/ANS-Me.

**Results:**

It was observed that the measurements for the inclination of the mandibular plane (SN.GoGn) in mouth breathing children were statistically higher than those in nasal breathing children. The posterior facial height was statistically smaller than the anterior one in mouth breathing children (PFH-AFH ratio). Thus, the upper anterior facial height was statistically smaller than the lower facial height (UFH-LFH ratio).

**Conclusion:**

We concluded that mouth breathing children tend to have higher mandibular inclination and more vertical growth. These findings support the influence of the breathing mode in craniofacial development.

## INTRODUCTION

The influence of respiratory function in development of orofacial structures has been widely discussed. According to Moss's Theory of Functional Matrix (Moss^1^, 1969), nasal breathing allows proper growth and development of the craniofacial complex interacting with other functions such as mastication and swallowing (Prates^2^ et al., 1997). This theory is based on the principle that facial growth is closely related to functional activity represented by different components of the head and neck region.

Nasal obstruction, however, leads to mouth breathing resulting in change of the tongue's position and half opened lips (Linder-Aronson^3^, 1970; Principato^4^, 1991; Proffit^5^, 1993). Therefore, any occlusion in the upper airways whether due to malformation, nasal mucosa inflammatory reaction (rhinitis), nasal septum deviation or Waldeyer's ring hypertrophy will result in nasal obstruction forcing the patient to breath through the mouth (Weckx & Weckx^6^, 1995). If we consider the doctrine of functional matrixes, the obstruction of nasal and ororespiratory airways may have impact on growth orientation of facial skeleton structure (Subtelny^7^, 1975).

The child with chronic mouth breathing, whether due to nasal obstruction or not, develops several morphological disorders during growth phase resulting in unfavorable dental-facial complex development (Linder-Aronson^3^, 1970; Shendal^8^ et al., 1976; Hulcrants^9^ et al., 1991).

Although there is significant evidence that poor nasal breathing will lead to mouth-nasal breathing its impact in dental facial growth is still unclear (Warren^10^, 1990).

Other authors disagreed from the statement that facial morphology and breathing mode are closely related (Warren^10^, 1990; Tourné^11^, 1991; Tourné & Scheweiger^12^, 1996).

These facts show clear need for further investigation about the impact of mouth breathing on dental facial growth and development at an early age. Therefore, this study will evaluate the morphological pattern of the face through side cephalometric radiographs in order to report existing differences between nasal and mouth breathing pediatric patients.

## OBJECTIVE

The objective of this study was to evaluate through cephalometric analysis the differences in facial proportion between mouth breathing children and normal breathing pediatric patients.

## MATERIAL AND METHOD

First this study was submitted and approved by the Ethics Committee of the Dental School, Ribeirão Preto- USP under # 2003.1.372.58.1. Sixty pediatric patients aged 6 to 10 years underwent otorhinolaryngological evaluation to diagnose the type of breathing mode in the outpatient center of Otorhinolaryngology of the Clinical Hospital, Medical School, Ribeirão Preto- USP, with history of patients and otorhinolaryngological examination (oroscopy – Brodsky & Kock^13^, 1992; anterior rhinoscopy; otoscopy and lateral skull radiograph – Cohen & Konak^14^, 1985) and data were recorded in a previously designed protocol.

Pediatric patients were divided into two groups: Group I with mouth breathing children with severe airways obstruction used as experimental group, and Group II with nasal breathing children as control group. The two selected populations did not have previous history of nasal respiratory complex surgery or orthodontic treatment.

After selecting the two groups with nose and mouth breathing children patients underwent orthodontic evaluation through lateral cephalometric radiography. During radiographic procedure lead apron was used to protect patients. The same technician using the same device performed the exam under standardized technique.

Contour tracings were performed for dental facial anatomic structures and soft tissues of concern for the purposes of this study.

**Figure 1:**
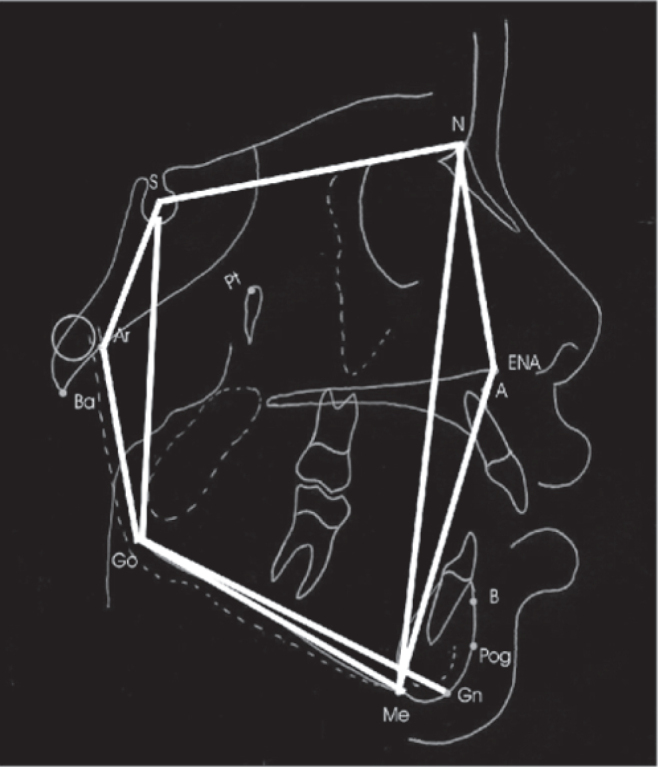
Angles and measures traced in this study.

Cephalometric points marked in cephalograms were as follows: (Figure 1)

• (S) Sella: Mid point of sella turcica;
• (N) Nasion: Most anterior point on fronto-nasal suture;
• **(A)** point: Position of deepest concavity on anterior profile of maxilla;
• **(Go)** Gonion: Most posterior inferior point on angle of mandible;
• **(Me)** Menton: Point located in the intersection between cortical external mental portion and cortical inferior mandible portion. Lowest point on the mandibular symphysis.
• (ANS) Anterior Nasal Spine – point located at the end of the anterior nasal spine;
• (Gn) Gnathion – most anterior and lowest point on the mandibular symphysis determined by bisectrix of the angle formed between the mandibular plane and a perpendicular line of it tangentially to the most anterior region of the symphysis;
• (Ar) Articulare – point located at the cross-section of posterior contour of the mandibular condyle with the occipital bone base.

After locating the landmarks of anatomical skeletal points, cephalometric angular and linear measurements obtained were as follows:

1) SN.GoGn Angle: determined by the intersection of S-N line with the mandibular plane (Go-Gn). Gives the inclination of the mandibular plane relative to anterior base of the skull.
2) ArGo.GoMe Angle (Gnathion Angle): determined by the junction of the ArGo line with GoMe line. Gives the degree inclination of the ramus relative to the mandible body.
3) N-Me Angle: linear measurement corresponding to the facial height of the anterior face.
4) N-ANS Angle: Represents the anterior upper height of the face.
5) ANS-Me Angle: Establishes the anterior lower height of the face.
6) S-Go Angle: Linear measure that gives the posterior height of the face.
7) S-Ar Angle: Gives the upper posterior height of the face.
8) Ar-Go Angle: Gives the lower posterior height of the face.

After performing cephalometric measurements the proposed indexes were as follows:

1- (iAF) Facial Height Index, obtained from the posterior facial height and total anterior facial height ratio. (iAF = S-Go/N-Me).
2- (iAFA) Index of Facial Height, obtained from the anterior lower facial height and total anterior facial height ratio. (iAFA = ANS-Me/N-Me).
3- Index of Anterior Facial Ratio (iPFA), obtained from anterior upper facial height and lower anterior facial height ratio. (iPFA = N-ANS/ANS-Me).

## RESULTS

Two groups were formed with 30 male and female patients each (Table 2), aged from 6 to 10 years as shown in Table 1.

**Table 2:** Gender distribution frequency of children in each group

Gender	Type of breathing				Total
	Nasal		Mouth		
	n^o^	%	n^o^	%	
F	16	53.3	23	76.6	39
M	14	46.6	7	23.3	21
Total	30	100.0	30	100.0	60

**Table 1:** Age frequency of patients in each group

Age (years)	Type of breathing				Total
	Nasal		Mouth		
	n^o^	%	n^o^	%	
6 — 7	4	13.3	7	23.3	11
7 — 8	13	43.3	12	40.0	25
8 — 9	8	26.6	8	26.6	16
9 — 10	5	16.6	3	10.0	8
Total	30	100.0	30	100.0	60

Table 3shows data related to mean values, standard deviation and p-value obtained from T Student test to compare the means of patients with nasal and mouth breathing. Statistically significant differences found (p < 0.05) between both groups in SN.GoGn, iAF and iPFA, that is, the values of mandibular plane inclination in mouth breathing children were statistically higher than those of nasal breathing children. Posterior and anterior facial height ratio and anterior upper and lower facial height ratio were statistically lower in mouth breathing children against the nasal-breathing children.

**Table 3:** Mean values, Standard Deviations and p-value of Student's T test in the nasal and mouth breathing children comparison.

	Nasal Breathing		Mouth		p-valUE
	Mean	SD	Mean	SD	
SN.GoGn	33.16	4.09	36.36	5.22	0.011*
ArGo.GoMe	131.73	4.62	134.03	5.72	0.092
IAF	0.62		0.60		0.014*
IAFA	0.57		0.58		0.084
IPFA	0.77		0.73		0.045*

Level of significance 5% (p < 0.05)

## DISCUSSION

Inadequate growth of dentofacial complex results from several genetic and environmental factors. The presence of mouth breathing in pediatric patients is a relatively common fact and may result in a series of changes of facial skeleton as well as in malocclusions (Aragão^15^, 1985).

The impact of nasal obstruction in facial and dental growth is quite controversial due to the criterion used to define mouth breathing, which is many times a subjective one. The lack of straightforwardness of these exams may result in incorrect diagnostics and consequently in inadequate treatment. In this study, the diagnosis of the type of breathing was made by oroscopy (Brodsky & Kock^13^, 1992), anterior rhinoscopy, otoscopy and lateral skull radiography (Cohen & Konak^14^, 1985). Thus, such otorhinolaryngological evaluation ensured correct diagnosis.

Some studies report that mouth-nose breathing is not necessarily deleterious to growth (Hinton^16^, 1986). If nasopharyngeal and oropharyngeal air space are reduced, patients with mouth-nose breathing mode have excessive postural responses which contribute to increased antero-lower facial development increasing mandibular plane inclination that may impact dentofacial development (Warren^10^, 1990; Tourne^12^, 1996). Craniofacial morphology and dental patterns are affected by mouth breathing sustained for long periods during high potential growth spurs (Principato^4^, 1991; Lyle^17^, 2000).

This study evidenced that mandibular plane inclination in mouth breathing children was higher than that of nasal breathing children. Kawashima^18^ et al. (2002) and Kerr^19^ et al. (1989) reported the same findings in younger children in pre-school age (3 to 6 years): mandible may be retrognathic and posteriorly inclined, particularly if the level of respiratory obstruction ranges from moderate to severe. This condition could determine the increase in anterior facial height due to clockwise mandibular displacement, showing a vertical growth pattern in older children (11 to 14 years) as reported by Yang^20^ at al. (2002).

The facial height index could be used to diagnose excessive or deficient vertical dimension, as an indicator of mandibular rotation during treatment. If anterior facial height is increased relatively to posterior facial height, there are some signs that the mandible rotates downwards and backwards (Horn^21^, 1992). In this study posterior and anterior facial height ratio (iAF) and anterior upper and lower facial height ratio (iPFA) were statistically lower in mouth breathing children, indicating proportionally lower posterior facial height than anterior facial height, and anterior lower facial height higher than upper facial height in these patients. This fact confirms the evidence that mouth breathing children present clockwise rotation of the mandible stimulating increased vertical growth of the anterior portion of the face relatively to the posterior portion of the face. Tourné^22^ (1990) highlighted the hypothesis that mouth breathing should be considered as the major etiological factor of induced excessive vertical growth. Ung^23^ et al. (1990) reported that mouth breathing, regardless of being analyzed through subjective perception, was associated with increased anterior facial height. Conversely, Smith & Gonzalez^24^ (1989) & Warren^10^ (1990) stated that it was difficult to judge if elongated face was cause or effect of increased nasal resistance.

Vig^25^ (1998) & Fields^26^ et al. (1991), however, suggested that causal association between nasal obstruction and facial growth in children seem to be of multi-factorial nature. Klein^27^ (1986) & Vickers^28^ (1998) did not report any conclusive evidence of the impact of mouth breathing on the development of more elongated faces which are resulted from different neuromuscular adaptations linked to a predetermined genetic pattern. Shintani^29^ at al. (1996) suggested that abnormal facial morphology found in mouth breathing patients could be influenced by genetic and environmental factors (upper airways obstruction).

Trotman^30^ at al. (1997) also observed posterior rotation of the mandible and reduced posterior lower facial height in children with pharyngeal and palate tonsil hypertrophy aged 3 to 13 years. This observation, however was obtained from a selected sample with diagnosis performed through pharyngeal and palate tonsil observation in lateral cephalometric radiographs and clinical records, therefore not a very accurate diagnosis of nasal obstruction.

In a study in which differences in both genders were assessed, Kawashima^31^ (2002) found that pre-school boys with respiratory disorder during sleep presented higher anterior lower facial height than girls. In spite of that, Vig^25^ (1998) recorded a significantly higher percentage of nose breathing among girls than boys.

Conversely, evidences that Gonion angle (ArGo.GoMe) present statistically different values in mouth breathing children and nasal breathing children evaluated in this study were not found. This result was not reported by Ahlqvist-Rastad^32^ et al. (1988), once they found increased Gonion angles in mouth breathing children if compared against nasal breathing children. Discrepant findings could be due to samples used in both studies, especially in regards of the children's age in the group evaluated by Ahlqvist-Rastad^32^ et al. (1988), which had a very large age range from 1 to 14 years. Additionally, the study was performed with higher sample size (122 pediatric patients) regardless of the fact the group was heterogeneous in terms of age. Such fact may mask results, since it involves different age groups and phases of facial growth. One of the aspects to be considered is the fact that fourteen-year old individuals have already definitive facial dimensions achieved, whereas ten-year old individuals have not undergone puberty growth, and may still present considerable modifications in facial morphology. This study evaluated children aged 6 to 10 years with increased frequency of individuals aged 7 to 9 years, which did not have their full potential growth yet. According to Defabjanis^33^ (2003), maxilla and mandible present considerable growth in size at the age of 12 in such way that 90% of deformities occur up to this time period.

Unanimous consensus was not found, however, data seem to suggest a correlation between respiratory impairment and dentofacial deformity. Regardless of the lack of total understanding, maintenance and establishment of nose breathing is a key factor for proper dentofacial growth and development.

## CONCLUSION

Mouth breathing children tend to present increased mandibular inclination, vertical growth pattern with changes in normal facial proportions, characterized by increased anterior lower facial height and decreased posterior facial height in mouth breathing children, therefore evidencing the influence of respiratory function in craniofacial development.
